# Supplementary material for: All-optical spin switching probability in [Tb/Co] multilayers
Source: Sci Rep. 2021 Mar 22;11:6576. doi: 10.1038/s41598-021-86065-w (PMC7985155; doi:10.1038/s41598-021-86065-w)
Supplement: Supplementary file 1 — Supplementary Information. [file 41598_2021_86065_MOESM1_ESM.pdf]

Supplementary Information
All-optical spin switching probability in [Tb/Co] multilayers

L. Avilés-Félix[1,*], L. Farcis[1], Z. Jin[1], L. Álvaro-Gómez[1], G. Li[2], K. Yamada[2], A. Kirilyuk[3], A. V. Kimel[2], Th. Rasing[2], B. Dieny[1], R. C. Sousa[1], I. L. Prejbeanu[1], and L. D. Buda-Prejbeanu[1,+]
[1]Univ. Grenoble Alpes, CEA, CNRS, Grenoble INP, IRIG-SPINTEC, 38000 Grenoble, France
[2]Radboud University, Institute for Molecules and Materials, Heyendaalseweg 135, 6525, AJ Nijmegen, The Netherlands.
[3]FELIX Laboratory, Radboud University, 7 Toernooiveld, 6525, ED Nijmegen, The Netherlands
[*]lavilesf@cab.cnea.gov.ar
[+]liliana.buda@cea.fr

S1. Schematics of the sample composition

The general structure of the multilayered sample is labeled as [Tb_n/Co_m], where the index *n* and *m* correspond to the number of monolayers of each element. We have considered a multilayered system consisting of 30 × 30 × 30 fcc cells with periodic boundary conditions. We also assumed that there are no size differences between RE and TM atoms. With this configuration, [Tb_1/Co_2] correspond to a single monolayer of Tb followed by 2 monolayers of Co, and [Tb_4/Co_8] for 4 monolayers of Tb followed by 8 monolayers of Co. This structure is repeated several times to form the 30 × 30 × 30 cells used in the atomistic simulation. In the figure S1 we show a schematic of the arrangement of the spins for [Tb_1/Co_2], [Tb_4/Co_8], [Tb_4/Co_8] with a soft, medium and strong intermixing and TbCo alloy with 66% of Co that were discussed in the paper.

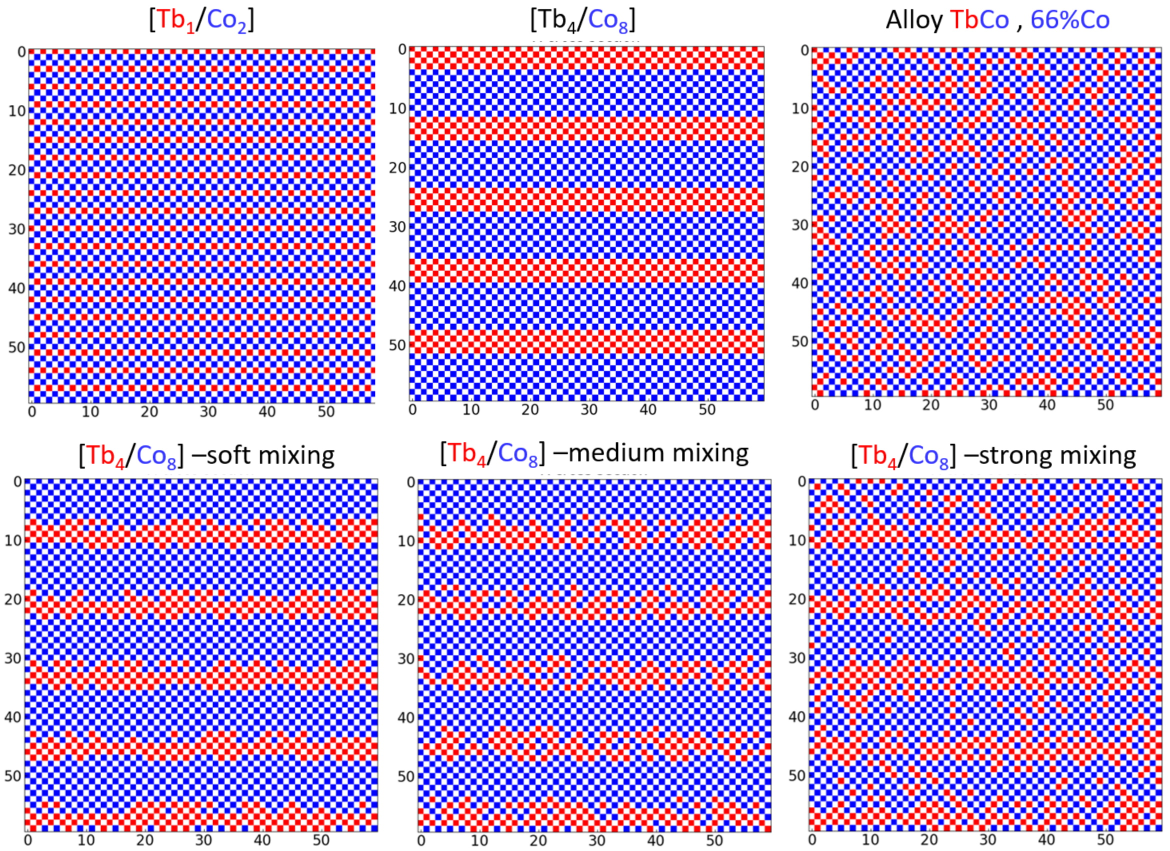

Fig. S1. Schematic of the different [Tb_n/Co_m] structures studied: [Tb_1/Co_2], [Tb_4/Co_8], [Tb_4/Co_8] with a soft, medium and strong intermixing and TbCo alloy with 66% of Co.

Following this type of structure is possible to have a clear view of the distribution of various spins in our samples and to determine the number of Tb-Co interfaces in each system.

S2. Normalized magnetization of Tb and Co sublattices in [Tb_1/Co_m] system

We obtained the normalized magnetization of Tb and Co as a function of the laser fluence for the multilayered system [Tb_1/Co_x], for x=1, 2, 3, 4 and 5. As shown on Fig. S2 only [Tb_1/Co_2] and [Tb_1/Co_3] present a clear window of fluence values in which thermally induced magnetization switching is observed. From these two systems we chose [Tb_1/Co_2] for the study of the stochasticity of switching.

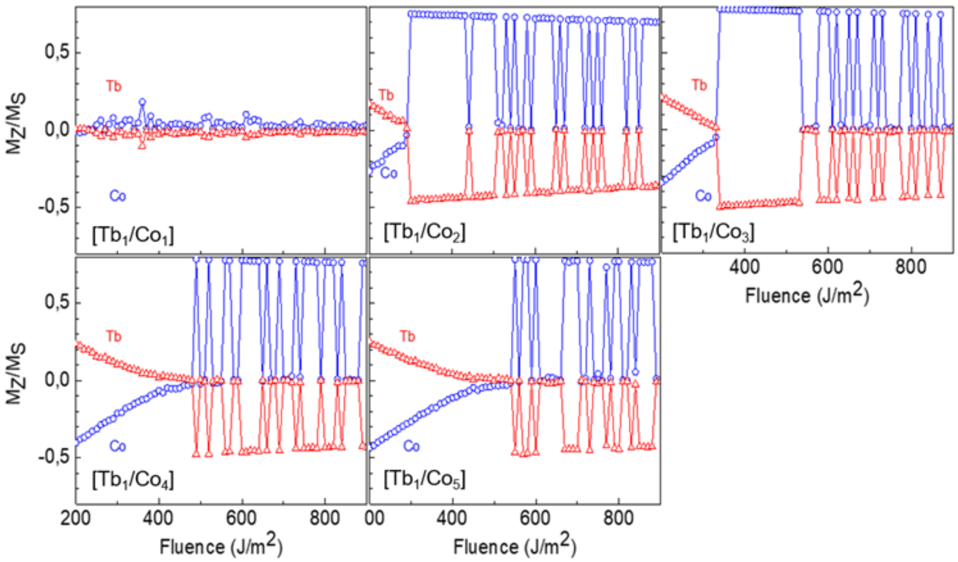

Fig S2. Normalized magnetization of Tb and Co sublattices as a function of the laser fluence. M_z/M_s ratios were extracted from the magnetization curves of the [Tb_n/Co_m] multilayers 20 ps after the single laser pulse has interacted with the system. A deterministic switching window appeared only in [Tb_1/Co_2] and [Tb_1/Co_3] multilayers.

S3. Dynamic magnetic properties of [Tb_4/Co_8] multilayers

We obtained the normalized magnetization of Tb and Co as a function of the laser fluence for the system [Tb_4/Co_8]. Fig. S3a suggest that, although there is not a clear window of fluences to observe thermally induced magnetization switching, the magnetic polarization of the multilayer is antiparallel to the initial state for all the fluence values. On the other hand, the magnetization dependence as a function of time in Fig S3b does not show a clear transient ferromagnetic state during the first ps after the laser heats the system as in [Tb_1/Co_2]. The zoom from Fig.S3c shows that the curves are very noisy around the origin which is an indication that the thermal fluctuation dominates the switching process instead of the exchange of angular momentum after demagnetization of the ferromagnetic layer. Moreover, only a partial reversal of the magnetization is observed, the magnetization distribution of the ferromagnetic layers ends up in to multi-domain state. Therefore, the normalized magnetization of Tb and Co in the multilayer [Tb_4/Co_8] does not follow the pathways as proposed in the model of Davies *et al*. (Davies, C. *et al*. Pathways for single-shot all-optical switching of magnetization in ferrimagnets. Phys. Rev. Appl. **13**, 024064, (2020)).

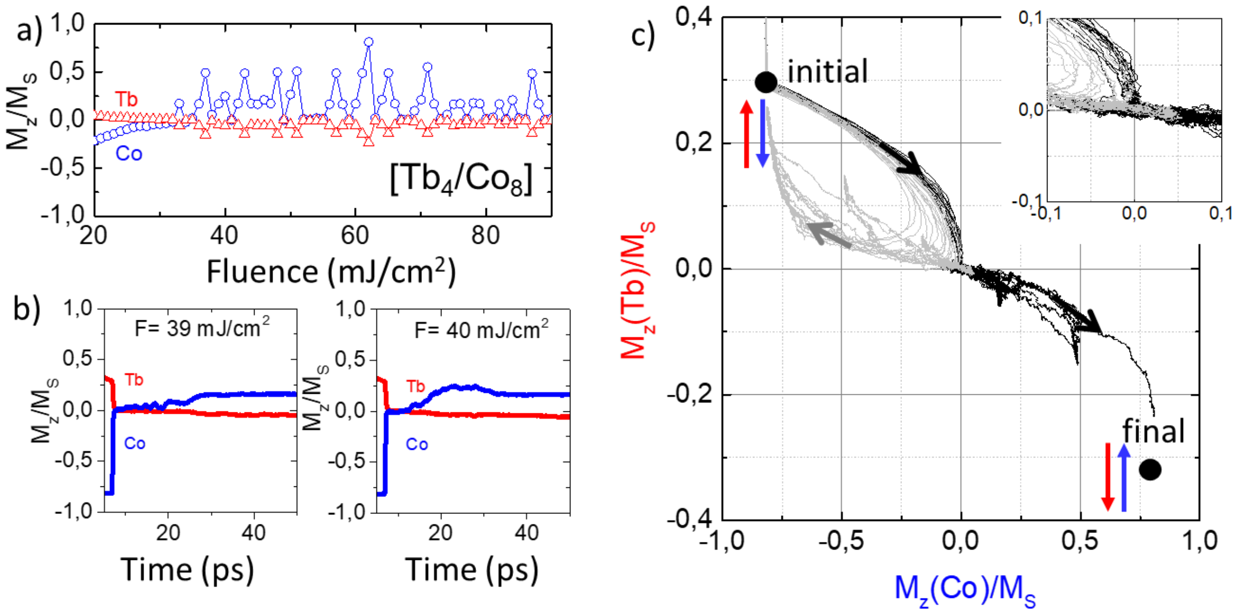

Fig S3. a) Normalized magnetization of Tb and Co as a function of the laser fluence. b) Time evolution of the normalized magnetization of Tb and Co, M_{Co} (blue) and M_{Tb} (red), after the application of a single laser pulse of 50fs at t = 7 ps in the system [Tb_4/Co_8] for two different fluences: 39 mJ/cm^2 (left) and 40 mJ/cm^2 (right). c) Magnetization phase diagram of the [Tb_4/Co_8] multilayer over all the simulated events.

S4. Intermixing of [Tb_4/Co_8] multilayers

In Fig. S4 we are showing the distribution of the Co and Tb spins for the ideal sample [Tb_4/Co_8] as well as that of the sample affected by the intermixing at the interfaces.

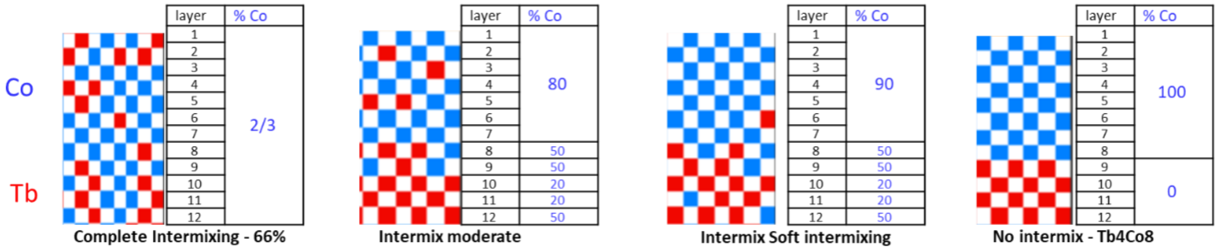

Fig. S4. Cross-section of sample [Tb_4/Co_8] with perfect interfaces and with intermediate (soft, moderate) mixing to the alloy with 66% Co. The percentage of sites occupied by the Co magnetic moments is given by each layer.

S5. Experimental evidence of stochasticity

In the experiments presented in the paper L. Avilés Félix *et al*. Sci. Rep. **10** 5211 (2020), we reported the helicity-independent magnetization switching of CoFeB/[Tb/Co]$_5$ multilayers, which have been demonstrated with a magneto-optical Kerr effect microscopy set-up. After testing the switching ability of our [Tb/Co]-based electrode, we continued with the integration of the optically switchable electrodes

within nano-sized magnetic tunnel junction structures. Although, we did not find proves of stochasticity in the [Tb/Co] in full-sheet films (the optical measurements were conducted and no specific measurements were intended to analyze the existence of rare events), we found some signatures of stochasticity in recent preliminary electrical tests of the Tb/Co-based electrodes within the magnetic tunnel junctions.

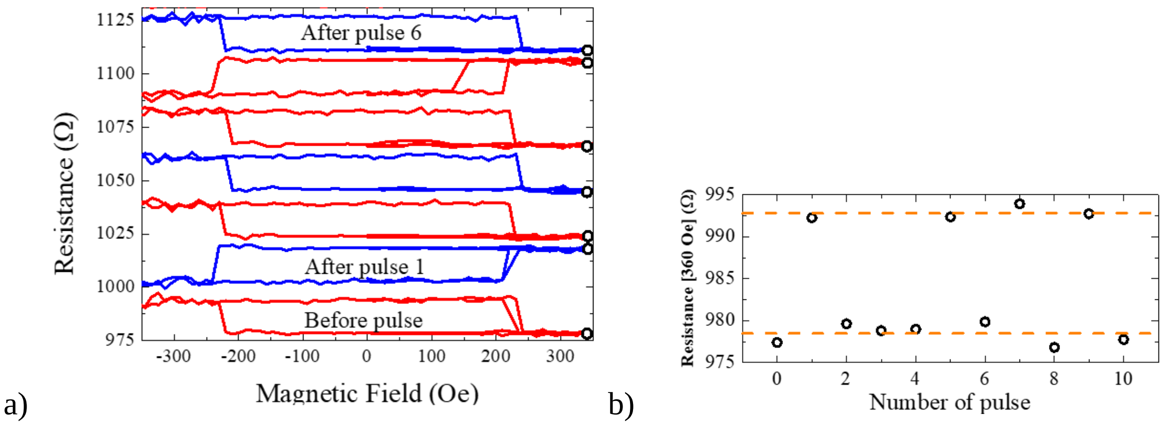

Fig. S5. a) Resistance loop of the nano-sized magnetic tunnel junctions consisting of CoFeB/MgO/CoFeB/[Tb/Co]$_5$. The resistance change corresponds to the resistance state of the CoFeB sensing layer. b) Value of the resistance of the magnetic tunnel junction at 360 Oe. – Unpublished results.

As you can see in Fig. S5, in experiments, which consist of measuring of the resistance loop after a series of single laser pulses on top of the CoFeB/[Tb/Co]$_5$ optically switchable electrodes, the inversion from the antiparallel to parallel state after a single pulse showed 8 out of 10 resistance loop inversion after each pulse. To further clarify and confirm that this observation corresponds to an experimental observation of the stochasticity found in the simulations, additional experiments need to be done.
